# CNVs in 8q24.3 do not influence gene co-expression in breast cancer subtypes

**DOI:** 10.3389/fgene.2023.1141011

**Published:** 2023-05-09

**Authors:** Candelario Hernández-Gómez, Enrique Hernández-Lemus, Jesús Espinal-Enríquez

**Affiliations:** ^1^ Computational Genomics Division, National Institute of Genomic Medicine, México City, Mexico; ^2^ Center for Complexity Sciences, Universidad Nacional Autónoma de México, México City, Mexico

**Keywords:** gene co-expression networks, breast cancer subtypes, copy number variations, conditional mutual information, luminal breast cancer, HER2+ breast cancer, basal breast cancer

## Abstract

Gene co-expression networks are a useful tool in the study of interactions that have allowed the visualization and quantification of diverse phenomena, including the loss of co-expression over long distances in cancerous samples. This characteristic, which could be considered fundamental to cancer, has been widely reported in various types of tumors. Since copy number variations (CNVs) have previously been identified as causing multiple genetic diseases, and gene expression is linked to them, they have often been mentioned as a probable cause of loss of co-expression in cancerous networks. In order to carry out a comparative study of the validity of this statement, we took 477 protein-coding genes from chromosome 8, and the CNVs of 101 genes, also protein-coding, belonging to the 8q24.3 region, a cytoband that is particularly active in the appearance of breast cancer. We created CNVS-conditioned co-expression networks of each of the 101 genes in the 8q24.3 region using conditional mutual information. The study was carried out using the four molecular subtypes of breast cancer (Luminal A, Luminal B, Her2, and Basal), as well as a case corresponding to healthy samples. We observed that in all cancer cases, the measurement of the Kolmogorov-Smirnov statistic shows that there are no significant differences between one and other values of the CNVs for any case. Furthermore, the co-expression interactions are stronger in all cancer subtypes than in the control networks. However, the control network presents a homogeneously distributed set of co-expression interactions, while for cancer networks, the highest interactions are more confined to specific cytobands, in particular 8q24.3 and 8p21.3. With this approach, we demonstrate that despite copy number alterations in the 8q24 region being a common trait in breast cancer, the loss of long-distance co-expression in breast cancer is not determined by CNVs.

## Introduction

Regulation of gene expression involves several processes by which the information contained in the genome is transformed into proteins. These processes within eukaryotic cell include signaling, chromatin remodeling, covalent histone modification, and transcription initiation, among others. Impairing of those processes are fundamental for the development of cancer, promoting tumor growth, cell proliferation, angiogenesis, or evasion of the immune response (Bian et al., 2022).

According to the World Health Organization, in 2020 around 685,000 people died from breast cancer (World Health Organization, 2020). It is the fifth cause of death from cancer. However, it is the first place in new diagnoses, with 2.26 million. This apparent contradiction between cases of death and those detected for breast cancer is largely explained by early detection (more than 90% of breast tumors are detected without metastases) and the relative good knowledge of the disease. Although breast cancer patients have a survival rate greater than 80% 5 years after diagnosis, this depends on the subtype.

The most commonly used classification system for breast cancer is the PAM50 gene expression signature, which divides breast cancer into four main subtypes: Luminal A, Luminal B, HER2+ and Basal-like subtypes Perou et al. (2000). The molecular classification of breast cancer arises from advances in genomic sequencing, taking into account the gene expression signature of 50 genes relevant for the disease. This classification has allowed a better evaluation, diagnosis and treatment of breast cancer.

The luminal A subtype is the most diagnosed breast cancer (between 40% and 50% of all cases) and is also the one with the best prognosis, with hormone receptor suppressors being a good therapy. The luminal B subtype is less common (between 20% and 30% of cases) but more aggressive, although with a good response to chemotherapy. About 15% of breast cancers have an overexpression of the Her2 gene and this makes them particularly aggressive. The worst prognosis is for patients with triple-negative cancer, which represent about 15% of diagnosed cases Tsang and Tse (2020).

Copy number variations (CNVs), refer to genomic changes that involve deletions or duplications of large DNA segments ranging in size from 1 KB to several megabases. Typically, a person has two copies of each gene inherited from their parents, but there are naturally occurring variations to this number. These genetic variants can include deletions, duplications, or insertions in the paternal or maternal chromosomes, or both, and are present in healthy individuals. In a more standardized definition, CNVs are stretches of DNA larger than 1 kb that display copy number differences in the normal or reference population (Scherer et al., 2007).

In cancer, CNVs can have a significant impact on gene expression and contribute to the development and progression of the disease, for instance, in oncogene amplification (Gajria and Chandarlapaty, 2011; Swain et al., 2023), tumor suppression gene deletion (Ried et al., 2019; Gupta et al., 2021), genomic instability (Duijf et al., 2019; Kalimutho et al., 2019; Neuse et al., 2020), or even drug resistance (Lim and Ma, 2019; Pös et al., 2021).

The 8q24 genomic region is a specific location on the long (q) arm of human chromosome 8. Amplifications and deletions of this region are involved in the development of certain types of cancer, such as prostate (Gu et al., 2020; Wilson and Kanhere, 2021), colon (Killian et al., 2006; Anauate et al., 2019; Nait Slimane et al., 2020), or bladder cancer (Kiltie, 2010). Research has identified several genetic variations within the 8q24 region that are associated with an increased risk of developing these cancers. In particular, the 8q24.3 region has previously been identified as one with significant activity in various types of cancer (Mahmood et al., 2014; Brusselaers et al., 2019; Ambele et al., 2020; Zheng et al., 2021), including breast cancer (Dorantes-Gilardi et al., 2021).

To analyze next-generation sequence data, contemporary biology often uses correlation networks to integrate the multiple sources of data. One of the most commonly implemented tools are the Gene co-expression networks (GCNs). GCNs are mathematical constructions based on the patterns of statistical correlation between genes across different phenotypes. These networks can help identify functionally related genes and pathways and provide insights into the underlying mechanisms of complex biological processes, such as cancer.

Previous studies found that the gene co-expression networks of cancerous samples differ significantly from those of healthy samples (Rai et al., 2017; Dorantes-Gilardi et al., 2021; Dorantes-Gilardi et al., 2020). In adjacent-to-tumor breast tissue, gene co-expression networks show a higher connection between genes from different chromosome, indicating coordination and cooperation between genes. However, this co-expression is dramatically lost in cancer GCNs, both when all subtypes are analyzed together (Espinal-Enríquez et al., 2017) and for subtype-specific GCNs (Alcalá-Corona et al., 2017; García-Cortés et al., 2020; González-Espinoza et al., 2021). The genes in cancerous samples tend to co-express mainly with their nearest neighbors and lose co-expression relations with medium and long distance genes. This phenomenon has been observed in lung cancer (Andonegui-Elguera et al., 2021), clear cell renal carcinoma (Zamora-Fuentes et al., 2020; Zamora-Fuentes et al., 2022), as well as other thirteen types of cancer (Garcia-Cortes et al., 2022).

The cause of the loss of co-expression in cancerous sample networks is still unknown. However, a general alteration in the transcriptional regulatory program could be underlying this effect. Therefore, assessing the influence that CNVs may exert on gene co-expression networks results appealing. In a previous work (Hernández-Gómez et al., 2022), we demonstrated that in Luminal B breast cancer molecular subtype, the copy number alterations of chromosome 8 influences marginally the gene co-expression landscape. Notwithstanding, the intrinsic heterogeneity of breast cancer molecular subtype could be differentially affected by CNVs, and concomitantly, the associated co-expression network.

Taking into account the previous studies that found differences in gene co-expression networks between cancerous and healthy samples, in this work, we proposed to analyze the influence of CNVs of the 8q24.3 region in the gene co-expression networks for each breast cancer molecular subtype. We analyzed the topological influence, the association of CNVs with network hubs, and the role of such hubs in a subtype-specific fashion.

## Materials and methods

Cancer and healthy samples were obtained from The Cancer Genome Atlas Consortium (TCGA) and preprocessed according to (Espinal-Enríquez et al., 2017). All samples were classified according to (Dorantes-Gilardi et al., 2021), resulting in 210 samples for Luminal A, 189 samples for Luminal B, 101 samples for HER2+, 215 samples for Basal, and 113 samples for normal adjacent-to-tumor tissues. The expression of 477 genes coding for proteins on chromosome 8 and the CNVs of 101 genes in the 8q24.3 region were analyzed for each sample.

We used the copy number alteration observed in chromosome 8 for the five phenotypes. A total of 101 CNVs were obtained using ascat data. For each of these CNV values, we constructed a CNV-specific gene co-expression network. To infer the conditional mutual information (CMI) for all phenotypes, we calculated as in (Hernández-Gómez et al., 2022), taking into account the co-expression between genes depending on the CNV values of chromosome 8 to observe the effect of variations in copy number on the co-expression of the entire genome. In this way, we obtained one network per CNV value, each of which can be considered a layer of a multi-CNVs co-expression network.

CMI calculations are thus the core of our analytic approach. In brief, CMI reflects the degree to which a random variable (here, the expression level *g*_*i*_ of a given gene *i*) is statistically dependent on another random variable (the expression level *g*_*j*_ of gene *j*) given a third random variable (the copy number landscape in the given genomic region *k*, *CNV*_*k*_) potentially affecting the relationship between *g*_*i*_ and *g*_*j*_.

*CMI*(*g*_*i*_, *g*_*j*_|*CNV*_*k*_) thus reflects the amount of information we have about the expression of gene *i* given our knowledge of the expression of gene *j* in the presence of copy number alterations in the region *k*. For the present case, *CMI*(*g*_*i*_, *g*_*j*_|*CNV*_*k*_) answers the following question: is the copy number landscape in the regions changing the way two genes are locally co-expressed or not?

To provide a statistically meaningful response to this question, it is necessary, nor only to provide systematic calculations of *CMI*(*g*_*i*_, *g*_*j*_|*CNV*_*k*_) for all the considered genes *i* and *j* and all the regions *k*, but also to perform rigorous hypothesis testing. To do this, we have resorted to the quite general and non-parametric, Kolmogorov-Smirnov test; since no assumptions need to be made in the nature of the probability distributions for gene expression nor copy number variants.

Hence, after constructing the networks, we calculated the Kolmogorov-Smirnov statistic to quantify differences between CMI layers. Once the CMI networks were constructed, we compared the number of intra-cytoband, inter-cytoband, and inter-arm *cis-*gene pairs for all chromosomes in the five phenotypes. We also evaluated the variations of these numbers depending on the CMI cutoff values and observed whether the intra-cytoband, inter-cytoband, and inter-arm numbers changed in accordance with the cutoff values.

Finally, we analyzed the most relevant genes in terms of their topological properties. We identified those genes that are both relevant for the structure and relevant for the proper function of a given phenotype.

### Conditional mutual information

Mutual information *I*(*X*; *Y*) is a measure of the mutual dependence between two random variables. It quantifies the amount of information that one random variable contains about the other. In other words, it measures the amount of reduction in uncertainty about one random variable given knowledge of the other random variable. Conditional mutual information, *I*(*X*; *Y*|*Z*), is the value of the mutual information between two random variables X and Y given (i.e., conditional to) the value of a third random value Z. Conditional mutual information measures the amount of reduction in uncertainty about one random variable given knowledge of the other random variable, but only in the context of a specific value of the third random variable.

$$I\left(X;Y|Z\right)=\underset{z\in z}{\sum }\underset{y\in Y}{\sum }\underset{x\in X}{\sum }p\left(x,y,z\right)\log\left(\frac{p_{z}\left(z\right)p\left(x,y,z\right)}{p_{x,z}\left(x,z\right)\;p_{y,z}\left(y,z\right)}\right)$$ (1)

Where *p*(*x*, *y*, *z*) is the joint probability of *X*, *Y* and *Z*, *p*(*x*, *y*) is the joint probability of *X* and *Y* and so on. It is worth noticing that conditional mutual information can only provide information about the dependence between the random variables, and cannot provide information about the causality between them. Conditional mutual information calculations in this work were made with the infotheo library of the R programming language (Meyer and Meyer, 2009).

### Kolmogorov-Smirnov test

The Kolmogorov-Smirnov (KS) test is a statistical test used to determine whether a sample of data comes from a known distribution. It is a non-parametric test, meaning that it makes no assumptions about the form of the distribution of the data. The test compares the empirical cumulative distribution function of the sample data to the cumulative distribution function of the known distribution, and quantifies the (maximal) difference between the two. If the difference is large enough, the null hypothesis that the sample data comes from the known distribution is rejected.

The KS test hence compares the cumulative distributions *F*_1_ and *F*_2_ of two probability functions *f*_1_ and *f*_1_ by quantifying the K-S statistic, defined as

$$D_{n,m}=\sup_{x}|F_{1,n}\left(x\right)-F_{2,m}\left(x\right)|$$ (2)

The null hypothesis is rejected (at significance level *α*), whenever:

$$D_{n,m}>c\left(\alpha \right)\sqrt{\frac{n+m}{n\cdot m}}$$ (3)

where $c\left(\alpha \right)=\sqrt{-\ln\left(\frac{\alpha }{2}\right)\cdot \frac{1}{2}}$

In the present context the KS test is appropriate since the sample sizes are sufficiently large and the CMI distributions can be safely assumed to be continuous. All tests were done using the ks.test library of the R programming language.

## Results and discussion

Here we report the main results of analyzing the conditional mutual information distributions associating the pairwise co-expression of genes conditional on the copy number landscape of the respective regions. These are data-based probabilistic tools to assess to what extent gene co-expression is affected by the underlying CNV structure in the same samples.

### Copy number alterations in 8q24.3 do not influence gene co-expression in breast cancer

**FIGURE 1:**
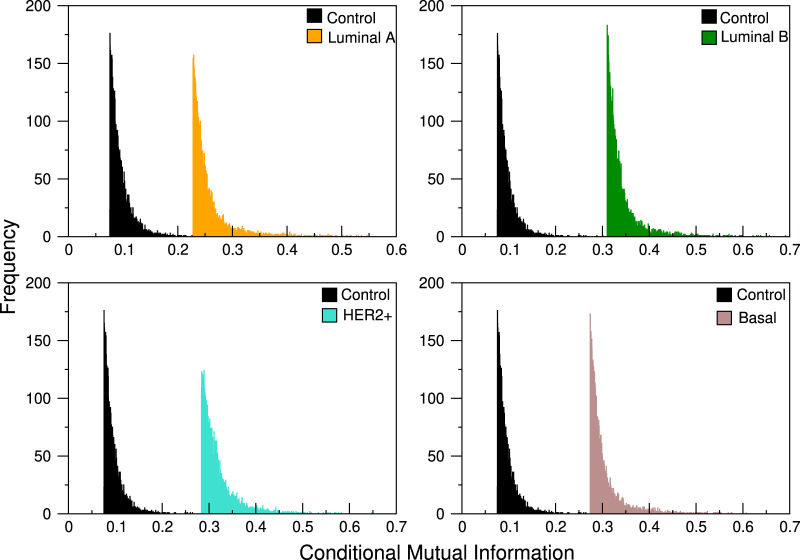
CMI comparison between control network and the four breast cancer subtypes. The abrupt cut in the left tail is due to the fact that we take, for each analysis, a pre-specified number of links, always staying with those that have the highest conditional mutual information values. In the distributions shown here, the cut selects only the first 3,500 links.

Each of the 101 genes of the 8q24.3 region for which the CNVs were used as the conditional variable in Eq. 1 producing 101 different distributions of CMI values, whose typical profile can be seen in Figure 1. Since the distributions suggest that the differences between them are minimal for all subtypes, we performed the K-S test in each case. For each subtype we take each of the 101 distributions and compare them with the remaining 100; since *D*_*n*,*m*_ = *D*_*m*,*n*_, we have 101 × 100/2 = 5,050 comparisons for each subtype. This is, we tested the null hypothesis that the distributions of CMI values are the same for each layer.

**FIGURE 2:**
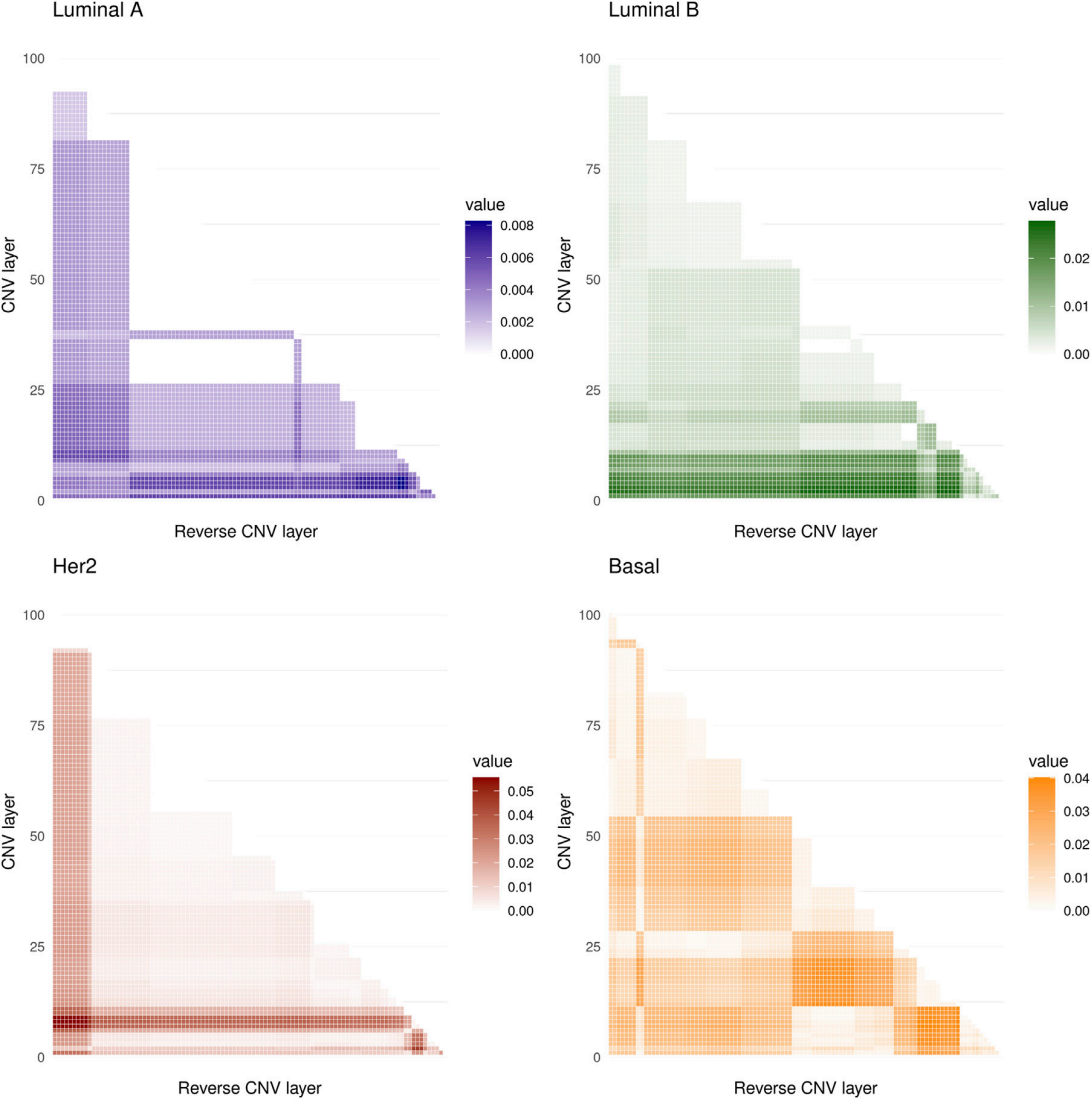
The heat maps show the values of the D statistic between the different distributions of the CMI values for the four molecular subtypes analyzed. 5,050 comparisons were made in all cases. The lowest values of D were obtained for the Luminal A subtype while the highest occurred in the Her2 subtype.

The values obtained are shown in Figure 2 where it can be seen that the maximum values of the *D* statistic for any subtype are low, the larger values are for the Her2 subtype and are approximately equal to 0.05, and the minima correspond to Luminal A, with values of around 0.008. Based on these values, we conclude that CNVs within the 8q24.3 region do not significantly affect the expression of genes located on chromosome 8.

It is worth noting that there is no significant variation between conditional layers for any of the phenotypes analyzed here. With this, we show that copy number alterations are not a significant factor altering the gene co-expression landscape in breast cancer or in healthy tissue in this region of chromosome 8.

Copy number variation is indeed one of several aspects that can influence (either individually or on a cooperative fashion) gene expression and co-expression patterns. Since we have been studying local gene co-expression phenomena and intuitively, one expects that the influence of CNVs on gene expression will also be predominantly local, we decided to perform a comprehensive analysis looking at all the pairwise co-expression relationships within chromosome 8, conditional on the full CNV variant landscape of the 8q24.3 region.

### Intra-chromosomal co-expression analysis

Intra-chromosomal gene co-expression refers to the simultaneous expression of genes that are located on the same chromosome. This means that they are physically close in the DNA sequence.

Intra-chromosomal gene co-expression can occur for different reasons. For example, genes that are physically close to one another on a chromosome may be regulated by the same regulatory elements, such as enhancers or promoters. This can lead to the coordinated expression of these genes.

The following results aim to present a broader view of this phenomenon in the context of breast cancer molecular subtypes.

**FIGURE 3:**
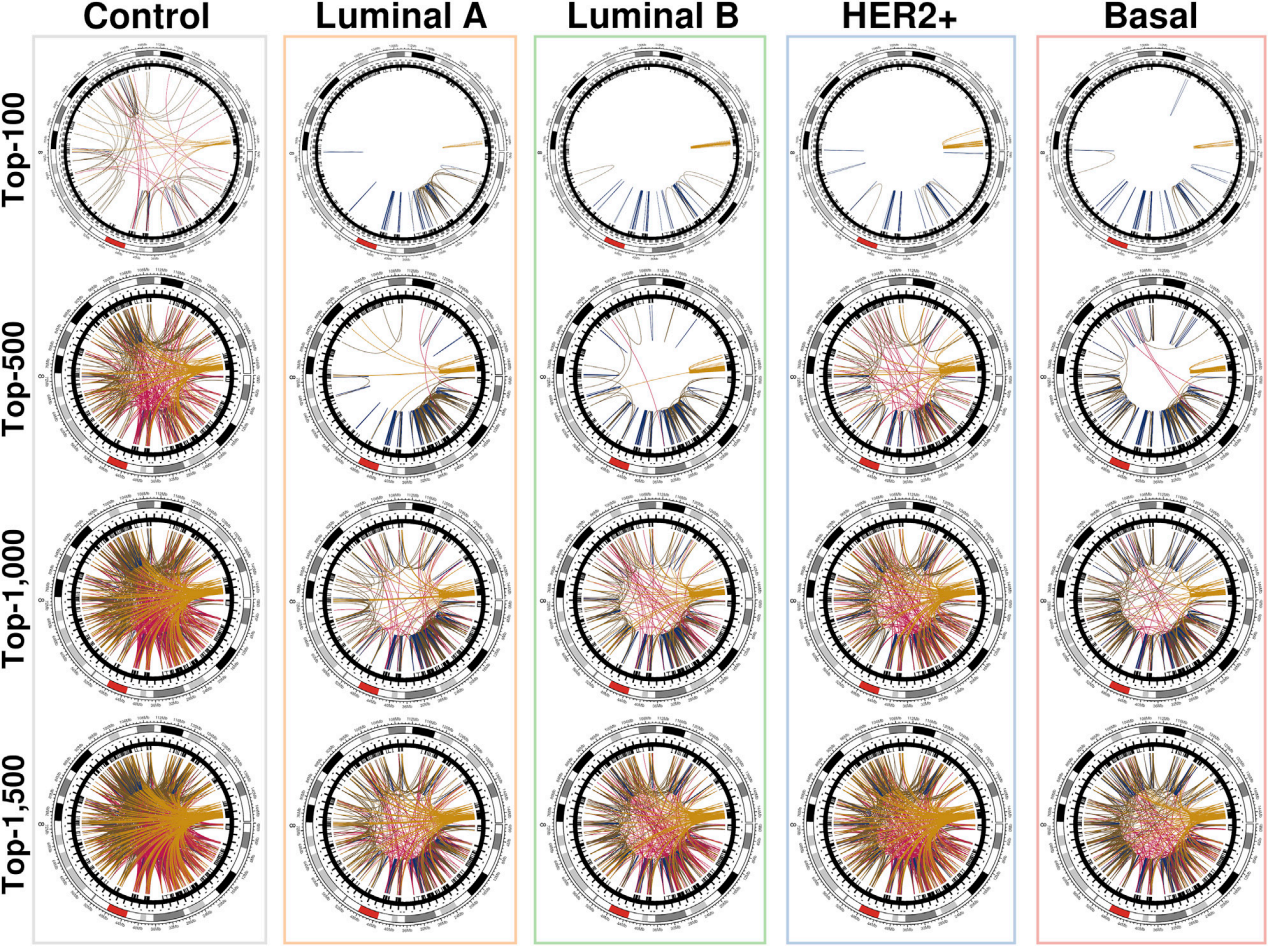
Top interactions of the five CMI networks at different cut-offs (100, 500, 1,000, and 1,500 edges). At first, intra-cytoband interactions dominate, mainly in q24.3, p21.3, p11.21 and p11.23; afterwards, inter-cytoband interactions (particularly in p-arm) grow, and finally, inter-arm edges arise. Red arcs at the external circle represents the centromere of Chr8. It can be clearly appreciated that for the normal tissue network, the distribution of interactions is remarkably more homogeneous than any cancer network, where interactions are preferentially located to neighboring regions. Circos plots were made with the R programming language package *circlize* (Cui et al., 2016).

The networks shown in Figure 3 were constructed using the first distribution and are representative of the behavior of all conditional layers. There, circos plots of gene co-expression interactions in chromosome 8 for the top-100, 500, 1,000 and 1,500 highest CMI values are depicted for all phenotypes.

**FIGURE 4:**
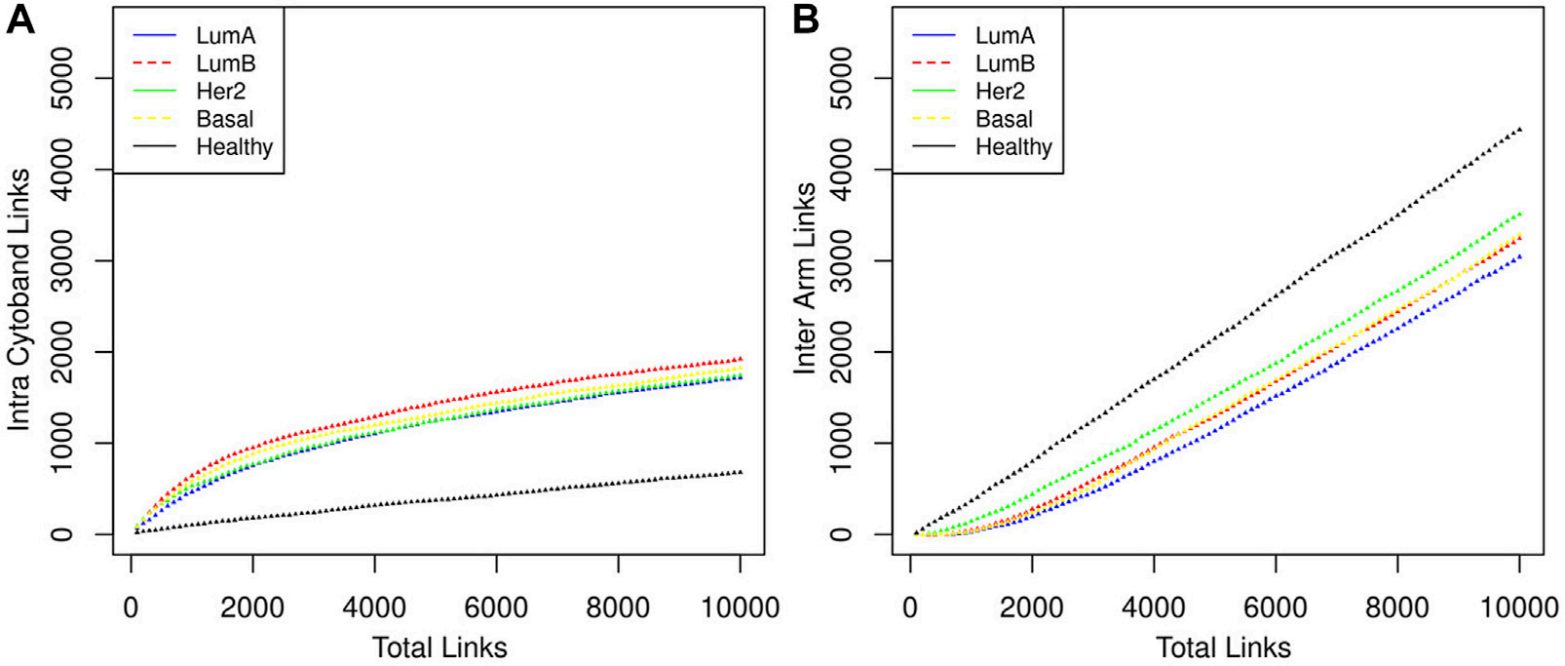
The distribution of the links in three categories is shown. **(A)** Intra cytoband. Co-expression with nearest neighbors is something that genes do in both healthy and cancerous phenotypes, although this tendency is markedly greater in the latter case. **(B)** Inter arm. In this category the behavior between the healthy phenotype and the cancerous ones is very marked, indeed Luminal B and Basal cases overlap throughout almost the entire range. It should be noted that the *order of appearance* of the links is determined by the magnitude of the CMI.

We can notice that Figure 3 is better understood when compared to Figure 4, which shows the cumulative growth of intra-cytoband and inter-arm links. By observing the growth line corresponding to the network of healthy samples as a reference, it can be seen how each subtype differs from the healthy reference network in terms of the growth of intra-cytoband and inter-arm interactions. Firstly, there is a lineal growth of intra-cytoband and inter-arm interactions in the healthy case, which is not the case of any breast cancer subtype. Additionally, all subtypes behave similarly in both panels, but with small differences. In Figure 4A, all breast cancer co-expression networks have a fast growth of intra-cytoband links, which is inversely proportional to the slow increase in the inter-arm edges.

In (García-Cortés et al., 2020) we demonstrated that the loss of inter-chromosomal interactions in breast cancer is evident in all phenotypes. Furthermore, the intensity of this loss is in agreement with the *malignancy* of the subtype: the most remarkable difference with respect to the healthy tissue network was observed in the Basal subtype, followed by HER2, then Luminal B, and finally, the most similar behavior to the control phenotype was observed in Luminal A.

Despite the Basal subtype being the most aggressive and the one with the worst prognosis, in the particular case of Chr8 intra-chromosomal edges, the most different behavior compared with the healthy case is observed in the Luminal B network (red lines in Figure 4).

In Figure 4 the total number of links ranges from 1 to (the top) 10,000, which gives a good sample of the behavior we want to illustrate. Intra-cytoband links grow a lot in the first few hundred larger CMI values in cancers but then they tend to saturate (see change of curvature in the plots). Eventually, all possible links will be formed and the curves will reach their maximum value. Finally, all link saturation lines are well above/below (panels A and B in Figure 4, respectively) the behavior of the healthy phenotype, thus showing the deficit of links at long distances as previously reported.

**FIGURE 5:**
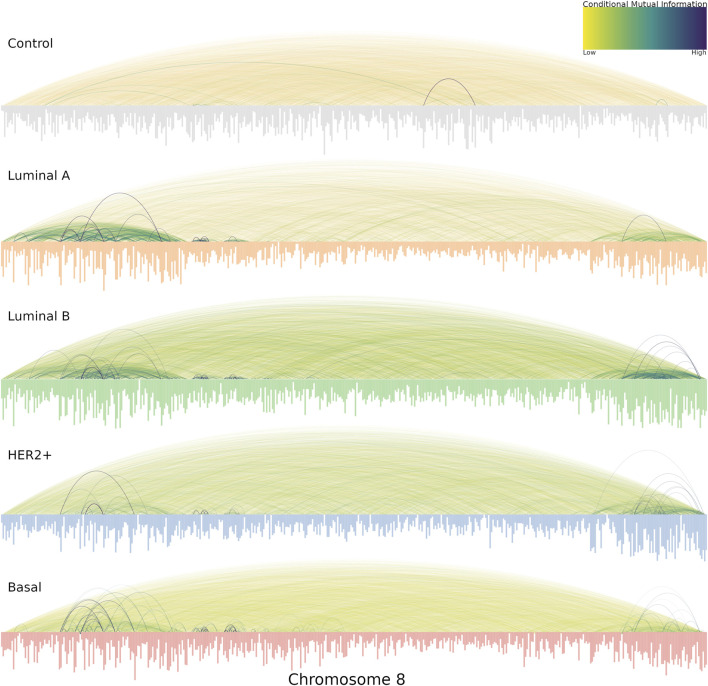
Chromosome 8 co-expression interactions for the five phenotypes are shown, with the genes placed according to its gene start position. The size of the genes is proportional to its degree. The color of co-expression interactions is related to the CMI values. Notice that for all cancer networks, the strongest interactions occur in the extreme places, in particular, 8p21 and 8q24.3.

Another relevant aspect that we noticed in the cancer chr8 gene co-expression networks is the location of highest co-expression values. This can be appreciated in Figure 5. In the case of the healthy network (labeled *Control* at the top) the vast majority of interactions present similar co-expression values, that is the reason for which several edges in the network present similar color. On the other hand, in the case of all breast cancer subtypes, highly dense regions of strong co-expression values are evident. Importantly, in all cancer cases, the q24.3 region contains a hotspot of strong interactions. Importantly, in all cancer cases, a hotspot of strong interactions is present in the q24.3 region. On the one hand, luminal networks present a large region from p23.3 to p11.23, while HER2 and Basal subtypes present a much more localized p-arm hotspot at p21.1-p21.3

Additionally, in the cancer networks, the degree of nodes is clearly higher than in their healthy counterparts. That is represented by the length of bars depicted below for each network of Figure 5.

Interestingly, the most connected genes in all cancer networks are located at 8p21.3, except in the HER2 network, where the most connected genes belong to the 8q24.3 region. Conversely, the healthy network’s most connected genes belong to q13 and q22 regions.

We have previously reported the appearance of a highly connected region located at 8q24.3 in breast cancer subtypes Dorantes-Gilardi et al. (2021). There, we showed that 8q24.3 is the only region in the entire genome in which all breast cancer subtypes present the same set of highly co-expressed interactions (top-100,00). That results is one of the motivations of this work, being focused in Chromosome 8. Here, we demonstrate that 8q24.3 is still important in terms of co-expression in breast cancer subtypes, but also 8p21 emerges as a relevant region.

In the case of HER2 network, it is worth noting that the HER2-enriched subtype was indeed named so, because of the amplification of a specific part of chromosome 17. In this work, we observe that 8q24.3 and 8p21.3 regions are also important but they do not depend on the copy number alterations, such as the case of amplification of 17q12 region, which is also related with global genomic instability (Ellsworth et al., 2008).

We want to stress that all of these results were obtained with TCGA-derived data. Further research must include other datasets in order to corroborate that these results are consistent independently of the data source.

## Conclusions and perspectives

The main conclusions of this work can be recapitulated in the form of a summary of findings, as follows:

1. Copy number alterations in the 8q24.3 region do not significantly affect gene co-expression in chromosome 8. Therefore, the loss of long-distance co-expression must be triggered by a different mechanism.
2. Basal and Luminal B breast cancer subtypes have the most remarkable loss of long-distance co-expression in this region.
3. HER2+ subtype has a worse prognosis than Luminal B, however, Luminal B behaves more differently from the healthy tissue. Perhaps, Luminal B has another mechanism involved in the co-expression program and the observed behavior in the chromosome 8 co-expression network is a manifestation of that.

For our dataset, CNVs does not influence gene co-expression networks in breast cancer in this region. However, copy number alterations are known to affect gene expression at different levels. The loss of long distance co-expression is strongly maintained in all cancer phenotypes, but in a different intensity.

The analysis performed here has been implemented for breast cancer molecular subtypes. Another classification approaches such as the TNM system, which is based on the tumor progression, should be incorporated to broaden the implications of copy number alterations in terms of their role on tumor progression. Further research in this line must be addressed to evaluate other aspects of CNVs in breast cancer.

Finally, this kind of analyses using different omic-approaches will definitively enhance our perspective and understanding of complex diseases such as breast cancer. We can envision to perform similar analysis at a whole genome scale in the future, though this endeavor will imply a high computational burden due to combinatorial effects. However, it is necessary to determine whether or not the copy number alterations observed in cancer are associated with the appearance of the phenomenon of loss of long-distance co-expression.

## Data Availability

The raw data supporting the conclusion of this article will be made available by the authors, without undue reservation.
